# Postapproval clinical experience in the treatment of Dupuytren’s contracture with collagenase clostridium histolyticum (CCH): the first 1,000 days

**DOI:** 10.1007/s11552-014-9645-7

**Published:** 2014-05-31

**Authors:** Scott M. Schulze, James P. Tursi

**Affiliations:** 1 Delmarva Hand Specialists, 34434 King Street Row, Suite 2, Lewes, DE 19958 USA; 2 Clinical Development, Auxilium Pharmaceuticals, 640 Lee Road, Chesterbrook, PA 19087 USA

**Keywords:** Collagen, Collagenase clostridium histolyticum, Contracture, Dupuytren, Hand, Nonsurgical treatment

## Abstract

Dupuytren’s contracture is a benign fibromatosis of the palmar and digital fascia of the hand of uncertain etiology, resulting in nodules and cords beneath the skin of the palm of the hands that may lead to the development of contractures. Surgical intervention is often considered when metacarpophalangeal (MP) joint contracture is 30° or more, or when there is any degree of proximal interphalangeal (PIP) joint contracture. Collagenase clostridium histolyticum (CCH) is a nonsurgical, minimally invasive enzymatic drug indicated for the treatment of adult patients with Dupuytren’s contracture (DC) and palpable cord. CCH has been available for approximately 3.5 years, and postapproval experience indicates that the effectiveness of CCH is equivalent to or better than efficacy observed in clinical trials, as seen by lower injection rates to achieve clinical success. Postapproval experience has shown a risk-benefit profile that favors CCH for patients not indicated for surgery based on current recommendations and shows also that treating earlier-stage vs later-stage joint contracture results in significantly better outcomes on average. Postapproval surveillance reveals a safety profile similar to that observed in clinical trials. Nonserious adverse events are mainly local reactions; tendon rupture, a serious adverse event, is reported rarely in the clinical practice setting and at a lower rate than in clinical trials. Risk Evaluation and Mitigation Strategy (REMS) training is designed to mitigate benefit vs risk to achieve safe and effective use of CCH.

## Introduction

Dupuytren’s contracture (DC) is a connective tissue disorder of the hand in which the development of tissue abnormalities and subsequent contractures within the palmar fascia can cause flexion deformity of the affected fingers, subsequently limiting use of the hand. The disease may initially present as firm nodule(s) in the palm proximal to the metacarpophalangeal (MP) and proximal interphalangeal (PIP) joints and then can progress to form cords [44]. Additionally, nodules, skin pitting, dimpling, tenderness, and contracture of the MP or PIP joints may be present upon examination [17, 30, 44, 47]. Typically, the disease progresses slowly, and patients may put off consulting their physician until the degree of disability keeps them from performing work or recreational activities that require manual dexterity of the affected hand; Dupuytren’s contracture can also limit a patient’s ability to perform activities of daily living [47].

### Epidemiology

Dupuytren’s disease (DD) is thought to mainly affect older persons of northern European descent [44]. The prevalence of DD varies with age, population groups surveyed (e.g., geographic location, age, patient characteristics), and methods of data collection, with estimated prevalence rates ranging widely from 0.2 % (in the general population) to as high as 56 % (in subgroups of patients with factors associated with DD) [22]. DiBenedetti estimated an incidence of 3 cases per 10,000 people in a large US population survey, consistent with an earlier survey in a British population [17]. Bilateral disease appears to be more common in men than women [34]. Men typically present sooner than women, perhaps by as much as 10 years, and often present with more severe disease [47]. A familial component is recognized, with a pattern of inheritance suggesting an autosomal dominance with variable penetrance [8, 25].

### Treatment

Surgical intervention is usually considered when MP contracture is ≥30° or when there is any degree of PIP contracture [44, 47, 48]. The longer the deformity is allowed to progress, the greater the chance of contracture becoming irreversible [47]. This is especially true of PIP contractures, and therefore, they are often treated earlier and more aggressively. Historically, partial, regional, or limited fasciectomy (excision of the contracture) has been the most widely used surgical procedure among hand surgeons because it is associated with a lower rate of recurrence than fasciotomy (severing without excision) [44]. Needle aponeurotomy is a minimally invasive technique performed under local anesthesia and does not typically require formal postoperative hand therapy [5]. Although a common procedure in France, a US survey estimates about 12 % of patients receive needle aponeurotomy [17, 42, 44].

Nonsurgical treatment in early DC can obviate surgical risks [44]. Collagenase clostridium histolyticum (CCH) is a minimally invasive, injectable enzymatic treatment approved by the FDA in February 2010 for adult patients with DC and a palpable cord [4]. CCH injected locally lyses and weakens the collagen cord and leads to cord rupture, either spontaneously or after a standardized finger extension/manipulation procedure [4]. Advantages of this technique include no need for hospitalization, surgery, or general or regional anesthesia; prompt healing with generally localized adverse reactions; low pain intensity of short duration; low risk of infection or other wound healing complications; and little if any need for traditional hand therapy [15, 21, 26, 31]. Since its introduction, utilization of CCH has grown from about 5 % of all procedures for Dupuytren’s contracture in 2010 to about 30 % in late 2013, which is generally correlated with a decrease in percent of surgical procedures during the same period. This procedure may be considered in patients who are not yet candidates for surgical intervention or who are considering surgical intervention but can potentially benefit from a less invasive option.

The objective of this review is to provide practitioners with a comprehensive update of current published and presented data regarding the clinical efficacy and postapproval effectiveness of CCH. In April 2013, we performed a PubMed search using the search terms “Dupuytren” and “collagenase” to identify key publications then refined the search to also identify relevant postapproval data. In addition, a search was performed of abstracts/presentations from 2010–2013 annual meetings of the American Society for Surgery of the Hand and the American Association for Hand Surgery. The search criteria included level 1–4 data, with particular emphasis on efficacy, adverse events (AEs), recurrence, patient/physician satisfaction, and cost-effectiveness.

## Efficacy and safety of CCH in clinical trials

Experience with CCH is largely limited to clinical trial data. Although these data provide limited information about the effectiveness of CCH in the clinical practice setting, they provide information about important considerations for practice, including durability of response, treatment of multi-cord disease requiring multiple CCH procedures, as well as other outcomes such as quality of life (QoL) and patient satisfaction with treatment among patients who do not achieve reduction to 0°–5° contracture.

### Efficacy of CCH in pivotal clinical trials

**Fig. 1:**
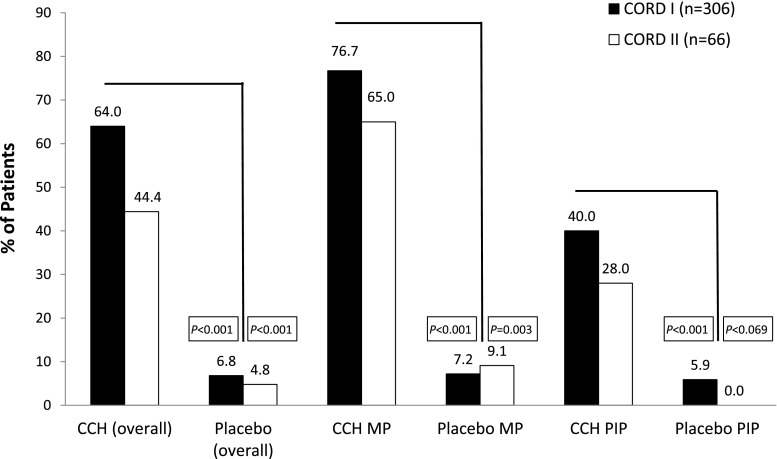
Percent of patients with reduction in contracture to 5° or less in overall primary, primary MP, and primary PIP joints after last injection in CORD I/II. A primary joint was defined as the first joint selected for injection; those treated second or third were considered nonprimary. Selection of a primary joint was determined by the investigator. All joints selected for injection had to meet the inclusion criteria for CORD I/II trials. *P* values are based on Cochran-Mantel-Haenszel test vs placebo, stratified by baseline severity and joint type. *CCH* collagenase clostridium histolyticum, *CORD* collagenase option for reduction of Dupuytren’s study, *MP* metacarpophalangeal joint, *PIP* proximal interphalangeal joint

The double-blind, placebo-controlled registration trials of CCH (CORD I and CORD II) showed that CCH was effective and well tolerated in adult patients with a palpable cord and MP contracture of 20°–100° or PIP contracture of 20°–80° [21, 26, 53]. The primary endpoint was clinical success, defined as reduction in primary MP or PIP joint contracture to 0°–5° 30 days after the last injection. Patients received CCH (0.58 mg per injection) administered at a maximum of three injections per cord in the double-blind phase, separated by a 30-day interval between injections. At 24 h after injection, patients returned to their treatment center for a standardized passive finger extension procedure that applied moderate pressure to achieve cord rupture (maximum 3 attempts) [26]. Both the injection technique and finger extension procedure have been previously described [26]. Most of the patients required one or two CCH injection procedures to achieve clinical success in the primary joint treated [2]. The rates of clinical success for the CCH and placebo in CORD I/II is illustrated for all primary, primary MP, and primary PIP joints in Fig. 1. In both trials, a greater percentage of patients treated with CCH compared to placebo achieved clinical success [2].

**Fig. 2:**
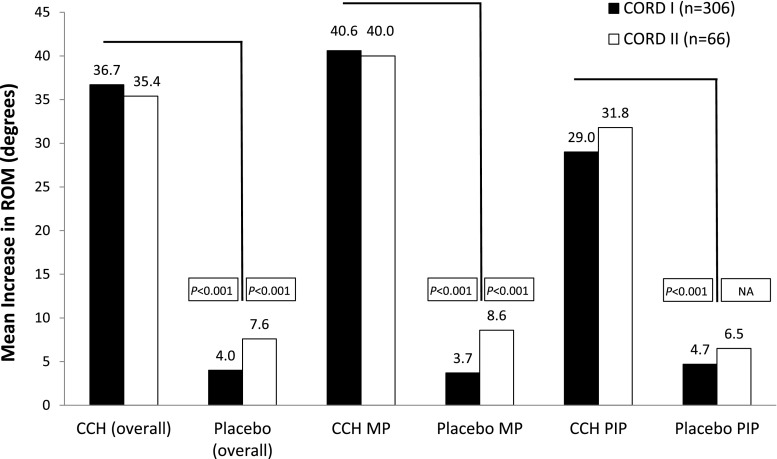
Mean change (improvement) in ROM from baseline to after last injection in overall primary, primary MP, and primary PIP joints in CORD I/II. A primary joint was defined as the first joint selected for injection; those treated second or third were considered nonprimary. Selection of a primary joint was determined by the investigator. All joints selected for injection had to meet the inclusion criteria for CORD I/II trials. *P* values are based on full factorial model analysis of variance (ANOVA) with treatment group, joint type, and baseline severity as factors. *CCH* collagenase clostridium histolyticum, *CORD* collagenase option for reduction of Dupuytren’s study, *MP* metacarpophalangeal joint, *NA* not applicable due to ineligibility for statistical analysis, *PIP* proximal interphalangeal joint

Range of motion (ROM) is frequently used as an assessment tool to evaluate the effect of surgical or other medical or therapeutic interventions in the clinical setting [18]. Mean ROM was significantly improved (*P* < 0.001) for all primary joints and primary MP joints in CORD I/II compared with placebo but not for primary PIP joints in CORD II (ineligible for analysis) (Fig. 2) [2]. A recent subanalysis of higher-risk patients included in CORD I/II showed no difference in efficacy or safety in CCH-treated patients by age, diabetes status, or sex, with 63 % of the patients reaching the primary endpoint of clinical success [41].

**Fig. 3:**
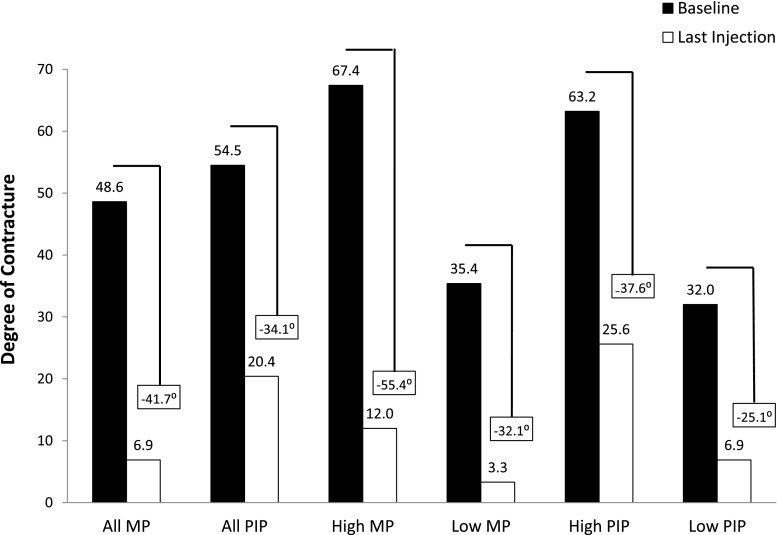
Mean degree of contracture and mean reduction in contracture of MP and PIP joints at baseline and after last injection by primary joints and high or low baseline severity. Individual cords could receive up to three injections; endpoint data are for last injection. MP low severity, ≤50° contracture; MP high severity, >50°; PIP low severity, ≤40°, PIP high severity, >40°. *MP* metacarpophalangeal joint, *PIP* proximal interphalangeal joint

Results from two open-label clinical trials (JOINT I and JOINT II) using the same treatment protocol as CORD I/II have also been published [53]. Overall, clinical success was achieved in 57 % (497/879) of MP and PIP joints, with an average of 1.2 injections per cord; by joint type this was 70 % (369/531) of MP joints and 37 % (128/348) of PIP joints. Less severely contracted MP and PIP joints (≤50° for MP and ≤40° for PIP joints) responded better than more severely contracted joints, which is consistent with the observation in the CORD I trial where joints with less severe contractures were also more likely to respond to treatment with CCH than were joints with more severe contractures [21, 26, 53], indicating that treatment when the contracture is less severe provides better outcomes when using CCH. This is clearly illustrated in a combined analysis of randomized, double-blind phase 3 trials, in which final degree of joint contracture was less for MP and PIP joints of less severe contracture than joints of high contracture [2, 19] (Fig. 3).

### Satisfaction with treatment

The CORD I/II pivotal data were examined to evaluate patient- and physician-reported satisfaction with CCH injection and to assess the value of a minimally invasive, nonsurgical intervention for DC [39]. Prior to treatment, patients were asked to rate the severity of DC on a 4-point scale: none, mild, moderate, and severe. At the end of the 90-day, double-blind period, patients rated their satisfaction with treatment using a 5-point Likert scale (very satisfied, quite satisfied, neither satisfied nor dissatisfied, quite dissatisfied, very dissatisfied) and improvement in contracture using a 100-point visual analog scale. Physicians independently rated initial contracture severity using a 4-point scale and change in contracture at the end of the double-blind period using a 7-point Likert scale.

**Fig. 4:**
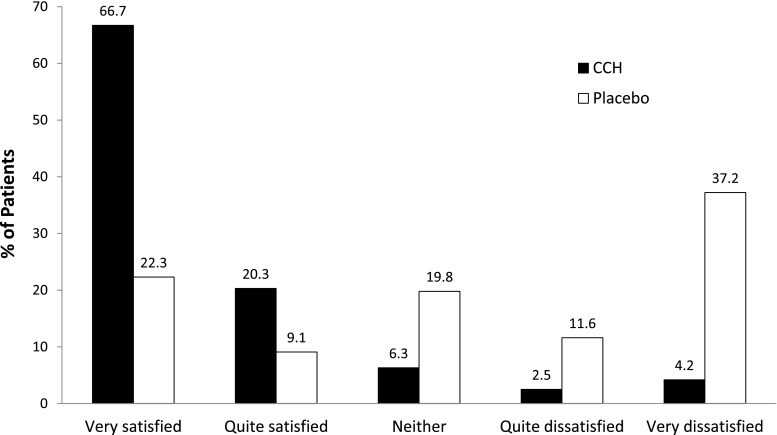
Patient satisfaction with CCH compared with placebo. *P* < 0.001 CCH vs placebo for all comparisons. *CCH* collagenase clostridium histolyticum

Data from 347 patients (249 CCH, 125 placebo) were included in the analysis. Patient-rated improvement and patient satisfaction were significantly higher among CCH-treated vs placebo-treated patients (*P* < 0.001 for both) [39]. Physician-rated disease severity was significantly lower (*P* < 0.001) and change from baseline was significantly better (*P* < 0.001) at study end for CCH-treated vs placebo-treated patients (Fig. 4). Notably, patient satisfaction ratings correlated with improved joint ROM after treatment (partial *r* = −0.29, *P* < 0.001 [controlling for treatment]). This correlation between satisfaction and improvement in ROM suggests that patient-rated satisfaction is related to improved ability to perform activities of daily living (ADLs) as well as better QoL, and therefore, improvement in ROM could represent a meaningful criterion for determining the effectiveness of treatment in a practice setting, which is not always clear solely from clinical trial goniometric criteria.

### Recurrence

Recurrence rates vary widely and are highly dependent on various factors. Becker and Davis conducted a systematic review of surgical interventions for primary DD [6]. The study evaluated the literature supporting individual procedures, focusing on data related to recurrence and complications to determine whether a particular technique appeared to be more favorable than others. The systematic review found major inconsistencies in reporting, with no agreed-upon definition of recurrence as well as inconsistencies for reporting the outcome of surgery, in that a variety of objective measurements were used to assess functional improvement. There was also disparity in the recording of complications, with only 62 % of papers quoting rates for specific complications. Werker et al. reported similar difficulties in reporting of recurrence, noting that rates of recurrence varied widely from 12 to 73 % for fasciectomy/aponeurectomy and 33 to 100 % for fasciotomy/aponeurotomy, mainly attributed to differences in the definition of recurrence and the duration of follow-up [52].

The Collagenase Option for Reduction of Dupuytren’s Long-term Evaluation of Safety Study (CORDLESS) trial is currently evaluating recurrence after CCH in a 5-year follow-up of patients initially treated in five previous clinical trials (CORD I/II, CORD I extension, JOINT I/II). Results from 3 years of follow-up have so far been published [37]. In this observational trial, recurrence was prospectively defined as ≥20° worsening of contracture in the presence of a palpable cord or medical/surgical intervention to correct the new or worsening contracture. Primary and secondary endpoints included recurrence in patients who had achieved clinical success (0°–5° contracture). Among the 643 patients treated in the original studies, 1,080 joints (648 MP and 432 PIP) were previously treated; among these 1,080 treated joints, 623 joints (58 %) had been successfully treated with CCH in the previous trials. Recurrence was seen in 35 % (217/623) of successfully treated joints. The study demonstrates that recurrence appears to occur with greater frequency in PIP joints than MP joints. The 3-year recurrence rate was 56 % for PIP joints and 27 % for MP joints that had achieved clinical success, and the authors observed that the least durable response occurred in high-severity PIP joints. Patients in CORDLESS had been previously treated with CCH for a maximum of eight injections per patient in the double-blind and open-label extension, and no additional AEs or long-term complications occurred as of the 3-year follow-up. There was no systemic allergic reaction to CCH for patients retreated for recurrence in CORDLESS.

Examination of mean contracture pattern over 3 years of interim follow-up in CORDLESS patients showed that those who had been successfully treated with CCH in the clinical trials and were nonrecurrent maintained improvement in contracture at a level similar to what had been achieved in clinical trials for MP contracture or slightly higher for previously treated PIP contracture [29]. The pattern of contracture showed progression in recurrent patients slowly over 3 years, but mean contracture still had not reached the pretreatment degree of contracture by 3 years. Over the 3-year interval, only 7 % (43/623) of patients who had been successfully treated in the trials had medical or surgical intervention [37].

### Durability of response

The durability of response to CCH was evaluated by Curtin et al. to identify factors that might predict durability [12]. Mean follow-up was 1,100 days from the first injection, and 94 % of patients had a 3-year visit. This analysis was based on the CORDLESS population consisting of 643 patients (1,079 joints). The predictors of durability for MP joints were unilateral disease, a total contracture index <110°, no prior surgical history for DC, and no family history of DD. For PIP joint contracture, low baseline severity was the only predictor of durable response, thereby supporting early intervention. In addition, patients who achieved full correction in the trials were less likely to have recurrence (≥20° increase in contracture or medical/surgical intervention) than those who had achieved partial correction of contracture with CCH.

### Earlier intervention

There are limited published data that show earlier intervention (i.e., when contracture is less severe) produces better outcomes. A retrospective chart review of 302 patients (61 earlier stage, 241 later stage) having only one joint treated with CCH in 2010 at 10 sites was conducted to determine if CCH treatment in earlier-stage DC joint contracture (≤30° [MP or PIP joints]) with a palpable cord resulted in a better contracture outcomes than later-stage disease (>30° contracture) with a palpable cord [49]. Final mean contracture angle was significantly better in joints treated earlier rather than later (earlier, 3.8° ± 6.9°; later, 14° ± 18.0°; *P* < 0.0001). Joints that were treated earlier showed an 85 % reduction in joint contracture compared to a 78 % reduction seen in joints treated later (*P* = 0.15). A limitation of this analysis is that study duration was not sufficient to assess if treatment of earlier-stage DC diminishes progression or recurrence.

In the double-blind phase of the CORD I/II clinical trials, patients could receive up to three injections of CCH to achieve clinical success. Blazer et al. retrospectively evaluated efficacy and safety outcomes in patients enrolled in CORD I/II who received CCH for a contracture >5° and <20° (i.e., patients who achieved this degree of contracture after the first injection and subsequently received another injection); outcomes for joints treated when contracture was >5° and <20° were compared with outcomes for joints treated when contracture was ≥20° [7]. The analysis showed that clinical success rates were higher for joints with contractures >5° and <20° (73 %, MP; 35 %, PIP) compared with contractures ≥20° (66 %, MP; 31 %, PIP). CCH may be an option to consider for less severe contractures and allow for earlier intervention in cases where surgical correction is delayed.

Correction of PIP contracture with CCH is associated with lower patient satisfaction than correction of MP contracture. In a retrospective chart review of 49 patients who received CCH for MP and PIP contractures, Couceiro et al. showed a significant difference in patient satisfaction related to degree of PIP correction [11]. There was a significant (*P* = 0.029) difference in patient satisfaction between those with PIP contractures who achieved a greater correction (mean = 37.7°) compared with those with less correction (mean = 8.9°). The difference in patient satisfaction was significant comparing MP and PIP contractures of the small finger (*P* = 0.00001), indicating overall that affected joint, digit, and degree of contracture correction have an important bearing on patient satisfaction with CCH treatment. Couceiro et al. concluded that postinjection manipulation of the small finger is more difficult with PIP than MP joint contracture after CCH. Thus, earlier intervention in PIP contractures with less contracture and possibly less finger manipulation, particularly for the small finger, may enable better outcomes in terms of patient satisfaction.

### Injections into multiple cords

Multiple joint contractures, whether in different hands or in the same hand, are common in patients with DC, and treatment of multiple contractures concurrently could potentially lead to increased complications. A pilot study showed that two cords could be concurrently treated with 0.58 mg of CCH per cord on the same hand, with efficacy and safety comparable to the treatment of a single cord [10]. Mean reduction in MP and PIP contractures were 30.3° and 22.1°, and mean increase in ROM were 30.0° and 17.1°, respectively, with no unexpected AEs. This study only treated a small number of patients but does suggest that two cords can be treated concurrently with efficacy and safety comparable to single cord treatment with CCH.

Coleman et al. reported on the results of a prospective, open-label, phase 3b study conducted in 60 patients at 8 centers, in which 0.58 mg of CCH was injected into each of 2 palpable cords in different fingers (*n* = 32) or same finger (*n* = 28) in the same hand at a single visit, followed by standardized finger extension procedure 24 h after CCH injection and 30-day postinjection efficacy and safety follow-up [9]. After a single injection of CCH per affected joint for each of two joints (i.e., two concurrent injections), the clinical success rate (0°–5° contracture) was similar to that seen in the CORD trials after a maximum of three injections [9, 21, 26]. Mean ROM increased from 50° at baseline to 82° at day 30 postinjection for MP joints and 51° to 78°, respectively, for PIP joints. Treatment-related local injection site AE rates were comparable to the single injection AE rates in CORD I/II, with higher rates for extremity pain, pruritus, lymphadenopathy, skin laceration, and blood blister. A pulley rupture from A2 through A4 and one tendon rupture in the right hand ring finger were reported, with no additional complications [9].

### Safety of CCH in clinical trials

#### Local adverse events

The overall clinical safety of CCH has been discussed at length in a published review [2, 16], based on data from clinical trials that included 2,630 injections in 1,780 cords in 1,082 patients with DC. Greater than 25 % of patients experienced local AEs such as edema (77 %), contusion (55 %), injection site pain (41 %), swelling (25 %), tenderness (29 %), and hemorrhage (35 %).

#### Immune-mediated reactions

Reported AEs potentially indicative of immune-mediated reactions include lymphadenopathy, lymph node pain, axillary pain, erythema, peripheral edema, and injection site pruritus. More than 85 % of patients developed anticollagenase antibodies after one injection of CCH, and nearly all patients developed antibodies after 3 injections, but with no correlation between antibody titer and absence or presence of a possible immune-mediated reaction or severity of such event [2, 16, 19].

#### Tendon rupture

In the CORD trials, three patients (0.3 %) had a flexor tendon rupture of the treated finger within 7 days of the injection and all three occurred after injections into the small finger [4, 16]. Hurst et al. reported that two tendon ruptures in CORD I that occurred in the small finger PIP joints and involved the flexor digitorum profundus and flexor digitorum superficialis and provided specific recommendations to reduce the potential risk of injection into PIP joints [24, 26].

## Effectiveness and safety of CCH in the practice setting

Three and a half years of experience after the introduction of CCH has enabled a better understanding of the effectiveness and utilization of CCH in clinical practice vs the results of clinical trials with their more rigorous methodologies. Variations in procedures and clinical experience in the practice setting may influence both safety and efficacy outcomes. Relevant factors include use of anesthesia, clinical experience with the injection and finger extension techniques, and how the pattern of use may be influenced by patient lifestyles and treatment expectations.

The effectiveness of CCH in the practice setting has been assessed by a chart review conducted at 10 US community and academic practice sites [40]. Data were based on 501 patients (74 % male; mean age 65 years), with 92 % (463/501) having sufficient data for analysis. The number of injections analyzed was 680 (422 MP, 258 PIP). Mean baseline contracture was 49° overall, 44° for MP joints, and 57° for PIP joints; mean posttreatment contracture was 12°, 8°, and 19°, respectively; and mean posttreatment ROM was 81°, 83°, and 77°, respectively. The practice setting showed a lower rate of injections per joint (1.08 for chart review vs 1.7 for clinical trials), and 93 % of joints received only one injection [40]. Local anesthesia postinjection before the finger extension procedure was adjunctive in 86 % (363/424) of cases (65 %, digit or metacarpal block only). The mean number of finger extension attempts per visit was 1.3, and only 18 % (77/424) of patients received finger extension beyond one attempt. A full release of cord was achieved in 67 % (284/434) of cases after the first injection (as compared to clinical success rate of 39 % in the CORD I trial after the first injection [26]) and was higher in MP (75 %) than PIP (48 %) of joints. The higher rate of full release after first injection in this chart review may possibly be related to the use of anesthesia during finger extension, less rigorous criteria for success than in the clinical trials, or that patients were generally satisfied with less than full extension. However, posttreatment contracture and ROM in this chart review were similar to CORD I/II but with fewer injections [40]. Fewer injections per joint, no requirement for physical hand therapy, and potentially fewer visits have important implications for health care resource utilization.

Similar findings of effectiveness also were shown by DeMarco et al. in a retrospective case series of 27 patients treated in rheumatology clinics [14]. The patients received 47 injections of CCH (0.58 mg) into 37 cords; 78 % (21/27) of patients met the inclusion criteria for CORD I/II. In contrast to the CORD trials, anesthesia was used in 72 % (34/47) of finger extension procedures, and ultrasound with high-resolution probe was used to guide CCH injection to avoid injury to the tendon sheath. Clinical success was achieved in 78 % (29/37) of all cords and in a higher percentage (93 %) of 27 cords that would have met CORD trial entry criteria. This compares favorably with 64 and 44 % achieving clinical success in the CORD I and CORD II trials, respectively. Procedural complications were hemorrhagic bullae (2), skin tearing (4), rash (1), lymphadenopathy (1), and axillary ecchymosis (1), which all resolved. As in the previous chart review, this rheumatology practice setting also showed a lower rate of injections per joint than the CORD trials (1.3 vs 1.7), and the higher clinical success rate might have been attributable to use of anesthesia.

### Postapproval safety surveillance

Ongoing postapproval surveillance of CCH safety is based on the MedWatch program, where AEs are voluntarily reported to FDA by patients and health care professionals and subsequently entered into FDA’s Adverse Event Reporting System (FAERS), which is accessible online. Peimer et al. analyzed the spontaneous reports received by the manufacturer in the first year after approval (from February 3, 2010 through February 2, 2011) and found that CCH’s safety profile was similar to that previously reported in clinical trials [38]. A total of 270 AE were reported in 115 patients, representing a drug exposure of approximately 5,400 injections. The most commonly reported AEs in the 1-year follow-up were skin laceration or tear (35 events), peripheral edema (30 events), and contusion (26 events). Most of the 35 reported skin tears healed without skin graft (2 patients) [38]. The analysis indicates lower reported rates of tendon rupture compared with controlled data from clinical trials. Tendon rupture in clinical trials was 1.14 per 1,000 injections [16], while the two tendon ruptures and one pulley injury in the spontaneous report represent a tendon rupture rate of 0.37 per 1,000 injections and a ligament injury rate of 0.15 per 1,000 injections [38]. A 30-month follow-up of postapproval safety has been reported for approximately 27,000 injections in 21,000 patients. Nineteen tendon ruptures and 3 ligament injuries were reported, representing a tendon rupture rate of 0.7 per 1,000 injections and a ligament injury rate of 0.11 per 1,000 injections. Thus, tendon rupture and ligament injury occured at a lower reported rate than in clinical trials, suggesting that the Risk Evaluation and Mitigation Strategy (REMS) training program has been effective. Tendon rupture following treatment of PIP contracture in clinical trials involved the small finger, and 3-year postapproval data showed greater risk in this digit; however, tendon rupture can occur in any finger or joint. One reason for this is that vertical (perpendicular) injection of CCH into the joint can result in missing the cord and injecting the tendon because there is limited space between cord and tendon. Proper injection technique is discussed in the “Practical Considerations” section below.

## Practical considerations

### Decision to treat

A systematic literature review of English and non-English articles on DC published from September 1, 1960 until December 1, 2010 was conducted using Medline, EMBASE, and the Cochrane Database of Systematic Reviews [46]. The review, which used American Society for Surgery of the Hand (ASSH) level of evidence criteria, considered factors that influence treatment decisions, such as number of fingers affected, type of joint, and severity of contracture, and also reviewed type of study, how the research question was determined, and level of evidence as a framework for analysis of the DC literature [1, 54]. A decision tree for treatment of DC was constructed based on available evidence-based medicine for the three primary modalities, namely, fasciectomy, needle aponeurotomy, and CCH. Sixty-six percent (191/289) of peer-reviewed papers identified consisted of case series or expert opinion, and only 14 papers qualified by ASSH criteria for level 1 or level 2 evidence (8 fasciectomy, 5 CCH, 1 NP). This review revealed that as more treatment options become available, decision-making is hampered by a limited number of high-level studies and that sufficient data from prospective, randomized, clinical trials to guide clinicians who treat DC remains an unmet need. Until such time, important factors that influence treatment decisions include number/type of affected joints, severity of contracture/rate of progression, surgical history, and patient-centric issues.

CCH can be used to treat contractures that interfere with hand function and cause functional disability affecting ADLs or patient lifestyles, provided that the patient has both a palpable cord and no comorbidities that may interfere with treatment [23]. In the CORD trials, patients had a palpable Dupuytren’s cord and at least 20° of MP or PIP contracture and were not able to simultaneously place the affected finger and palm flat on a table. MP contractures ≥30° and PIP contractures ≥20° usually result in a positive table-top test and interfere with hand function [23]. An additional consideration for utilization of CCH is a web contracture that interferes with grasp or pinch. There is no contraindication to the use of CCH, but comorbidities to consider include coagulation disorders, use of anticoagulant, or chronic muscular, neurologic, or neuromuscular disorders affecting the hand.

### Injection technique

The CCH injection technique was modified based on experience in the CORD I trial. It is recommended that the injection should be made with a 27-gauge, 1.25-mm (0.5 in.) needle. Cords of the little finger should be injected no more than 4 mm distal to the palmar crease and no deeper than 2 to 3 mm [26]. In addition, the needle should be placed into the PIP cord on a horizontal plane and not vertically. Stabilizing the needle while pushing the plunger also helps to prevent injection through the cord [24].

A REMS is a strategy designed to manage a known or potential serious risk associated with a drug or biological product. The REMS for CCH was developed to inform health care providers about the risks of tendon rupture, serious AEs affecting the injected extremity, and the potential risk of serious hypersensitivity reactions (including anaphylaxis) associated with CCH [3]. The REMS communication plan includes a training guide and video for health care providers who are likely to prescribe CCH, including hand surgeons, orthopedic surgeons, plastic surgeons, general surgeons, and rheumatologists. REMS provides clinicians with instructions for properly preparing and injecting CCH and using the proper finger extension procedure to achieve cord disruption. Enrollment consists of three steps: (1) reviewing the training materials; (2) completing, signing, and faxing the physician enrollment form to be able to order CCH; and (3) completing, signing, and faxing the site enrollment form to register site(s) for shipping.

### Finger extension technique

Skin lacerations are common and treatment-related lacerations occurred at a rate of 11 % in the clinical trials [16]. Risk of skin tears is greater as severity of contracture increases, and these lacerations occurred primarily in patients who had experienced severe baseline contracture over many years [27]. The cord can become adherent to the skin overlying the cord, which often becomes thin and more easily damaged [19]. Thus, over-strenuous postinjection manipulation should be avoided to reduce the risk of skin laceration, which can also interfere with fitting a good splint because of pain. In the clinical trials, skin lacerations healed without treatment and did not affect clinical outcome. In the published 1-year postapproval surveillance review, skin tear was reported at a rate of 6.5 per 1,000 injections [38].

Adherence to the recommended CCH postinjection standardized manipulation procedure is advised to reduce the risk of skin tear, especially in more advanced disease. The procedure for finger manipulation is also discussed in the REMS for CCH. Practitioner experience over time helps to reduce the incidence of skin tears, and follow-up care is very important in achieving the desired outcomes. Patients should be instructed to perform daily finger extension and flexion exercises during recovery and not to perform strenuous activity with the injected hand along with the proper, compliant use of nighttime splint.

### Spontaneous cord rupture

Spontaneous cord rupture has occurred after CCH injection. Gill et al. retrospectively reviewed 36 consecutive patients (43 joints) treated with a standard injection of CCH, followed by a 24-h return visit [20]. Average baseline MP and PIP contractures were 43.1° and 53.5°, respectively. Twenty-nine joints achieved full correction (20 MP, 9 PIP), and 7 patients had spontaneous cord rupture, resulting in full (4 patients) or partial correction (3 patients). Skin tear occurred in 3 of 36 patients. A recent MRI examination of cord structure in five patients who were treated with CCH and had no residual contracture after finger manipulation showed that CCH does not simply weaken the cord but actually causes disorganization and dissolution of the cord, which may help to explain the histologic basis for spontaneous cord rupture. MRI demonstrated discontinuity of the cord in all cases along with a significant reduction in cord volume from 670 to 188 mm^3^ and a significant increase in signal intensity (demonstrating disorganization of cord collagen) from 632 to 2,021, an average of 320 %, which together showed both significant reductions in diseased tissue rather than simple cord division and significant disorganization of cord collagen [35].

### Splinting after CCH

Following the finger extension procedure(s), patients should be fitted with a splint and provided instructions for use at bedtime for up to 4 months to maintain finger extension [3].

A recent study by Skirven et al. showed that treatment with CCH followed by gradual and progressive extension of the joint using a splint may be a more effective intervention strategy than passive extension used in the trials [45]. Twenty-one patients (22 fingers) with a mean passive PIP joint contracture of 56° received one injection of CCH. Contracture was reduced to 22° at time of cord rupture, followed by further decreases in PIP contracture to a mean of 12° 1 week later. Only 22 to 25 % of PIP joints in the CORD trial achieved clinical success compared with 55 % of joints in this study. Although local anesthesia (lidocaine) was not mandated by protocol in the CORD trials, it was used in this study and may have contributed to better results compared with the CORD trials. The use of anesthesia was particularly effective during cord rupture because it allowed patients to better tolerate the postinjection manipulation and led to fewer injections.

### Reimbursement

Because insurance coverage varies by payor and patient, health care providers should verify each patient’s health care benefits prior to initiating treatment. Individual plans may require a preauthorization evaluation. The general criteria include a letter of medical necessity, completion of a payor-specific prior authorization form, appropriate chart notes, history of past therapy and result, and a product information package insert. Medicare Part B patients do not require a preauthorization. Additionally, financial assistance may be available through the manufacturer for those patients who qualify.

### Cost-effectiveness

Health care decisions are based not only on efficacy and safety but also on economic considerations. Since the introduction of CCH, postapproval studies have been conducted to evaluate the cost-effectiveness of CCH. Malone and Armstrong used a Markov decision model to determine the comparative cost-effectiveness of CCH, limited fasciectomy (LF), and percutaneous needle fasciotomy (PNF) [33]. The study perspective was from that of a US payer. Patients were classified in the analysis based on clinical success after treatment, treatment failure resulting in revision, disease progression, and death. CCH was less expensive and associated with slightly more quality-adjusted life years (QALYs) than LF or PNF. Estimated mean costs over a 30-year period were US$4,489, US$18,345, and US$14,970 for CCH, LF, and PNF, respectively.

Ines et al. conducted a cost minimization study to estimate the cost of CCH versus fasciectomy in Portuguese patients with DC [28]. The direct costs and inpatient cost of surgery as well as postsurgical costs associated with patient follow-up visits and physiotherapy were estimated. The direct cost of CCH included vials used, administration of injection in an outpatient setting, and outpatient follow-up visits. The primary determinates of direct cost favoring CCH over surgery were reduction in cost of inpatient services and postsurgical physiotherapy, and the indirect cost favoring CCH was loss of productivity due to time out of work. The overall savings of CCH over surgery was 1,674€. There was a slight advantage related to direct costs, but the main advantage was related to the indirect cost of productivity loss.

Productivity loss has been studied by Naam in a recently published retrospective, longitudinal study of at least 2 years duration that compared CCH (*n* = 25) with fasciectomy (*n* = 21) [36]. Postprocedural follow-up averaged 32 months for CCH and 39 months for fasciectomy. Mean CCH postinjection contracture was 3.6° and 17.5° for MP and PIP joints, respectively, compared with 3.7° and 8.1° for fasciectomy, respectively. Patients treated with CCH returned to work at a mean of 1.9 days compared with 37.4 days after fasciectomy. There was no difference between groups related to type of work, no patient in the study met the criteria for recurrence (≥20° from time of joint correction), and no serious AEs were reported for either intervention.

Sau et al. conducted a cost-effectiveness study of CCH, LF, and PNF using a Markov model [43]. Of the three treatment interventions, the analysis favored LF, with CCH and PNF costing an average of US$1,844 and US$247 more than LF, respectively. The model is sensitive to cost of surgery, which may have variable outcomes related to postsurgical complications and associated costs. LF is the most frequently used procedure by hand surgeons in the USA, but a drawback of this procedure is a cumulative complication rate estimated to be as high as 19 % [50]. In addition, it is unclear whether recurrence was included in this model, a reported disadvantage of PNF, with an estimated recurrence rate as high as 85 % over 5 years [51].

An assessment of the direct and indirect costs of employees with DC compared with a matched cohort of non-DC employees showed that employees with DC had higher comorbidity rates, health care utilization, and work loss than non-DC employees. These factors resulted in increased mean health care costs of approximately US$4,227 for DC vs non-DC patients over a 9-year survey period [32], suggesting that the cost savings of early intervention should be weighed against the costs associated with prolonged, progressive DD.

De Salas-Cansado et al. conducted an analysis in Spanish patients to estimate the budget impact of CCH versus fasciectomy [13]. The analysis showed a difference in cost favoring CCH over fasciectomy for a typical case to be 1,030€, with a substantial difference in 3-year overall base-case budgetary impact of 8,835,750€ for fasciectomy and 3,819,591€ for CCH, suggesting that CCH can result in a substantial savings as a noninvasive outpatient procedure.

## Conclusions

CCH is a minimally invasive technique for treating Dupuytren’s contracture caused by collagen cords. It can be done in an office setting and is associated with mostly local AEs and short healing times compared with surgery. Clinical trials have shown that CCH is both effective and well tolerated and like surgery produces better responses in patients with less severely contracted joints. Postapproval surveillance has shown a safety profile similar to that seen in clinical trials and effectiveness equivalent to or better than that seen in clinical trials. A standardized finger extension procedure that includes local anesthesia helps reduce patient discomfort, and risks associated with CCH injection such as tendon rupture can be reduced through the use of the proper injection technique, a standardized finger extension procedure, and the REMS program developed to help physicians administer CCH safely.
